# Latest development in the synthesis of ursodeoxycholic acid (UDCA): a critical review

**DOI:** 10.3762/bjoc.14.33

**Published:** 2018-02-20

**Authors:** Fabio Tonin, Isabel W C E Arends

**Affiliations:** 1 Department of Biotechnology, Delft University of Technology, Van der Maasweg 9, 2629 HZ Delft, The Netherlands

**Keywords:** bile acids, biotransformation, hydroxysteroid dehydrogenases, production process, UDCA

## Abstract

Ursodeoxycholic acid (UDCA) is a pharmaceutical ingredient widely used in clinics. As bile acid it solubilizes cholesterol gallstones and improves the liver function in case of cholestatic diseases. UDCA can be obtained from cholic acid (CA), which is the most abundant and least expensive bile acid available. The now available chemical routes for the obtainment of UDCA yield about 30% of final product. For these syntheses several protection and deprotection steps requiring toxic and dangerous reagents have to be performed, leading to the production of a series of waste products. In many cases the cholic acid itself first needs to be prepared from its taurinated and glycilated derivatives in the bile, thus adding to the complexity and multitude of steps involved of the synthetic process. For these reasons, several studies have been performed towards the development of microbial transformations or chemoenzymatic procedures for the synthesis of UDCA starting from CA or chenodeoxycholic acid (CDCA). This promising approach led several research groups to focus their attention on the development of biotransformations with non-pathogenic, easy-to-manage microorganisms, and their enzymes. In particular, the enzymatic reactions involved are selective hydrolysis, epimerization of the hydroxy functions (by oxidation and subsequent reduction) and the specific hydroxylation and dehydroxylation of suitable positions in the steroid rings. In this minireview, we critically analyze the state of the art of the production of UDCA by several chemical, chemoenzymatic and enzymatic routes reported, highlighting the bottlenecks of each production step. Particular attention is placed on the precursors availability as well as the substrate loading in the process. Potential new routes and recent developments are discussed, in particular on the employment of flow-reactors. The latter technology allows to develop processes with shorter reaction times and lower costs for the chemical and enzymatic reactions involved.

## Introduction

**Figure 1:**
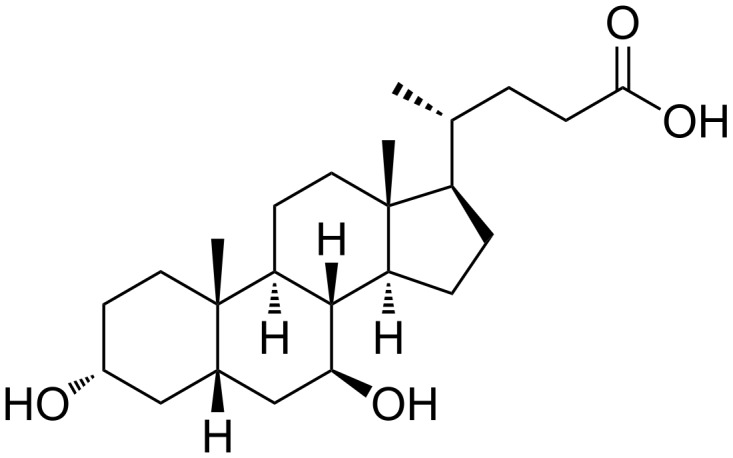
Chemical structure of UDCA.

Ursodeoxycholic acid (UDCA), is applied in the pharmaceutical industry (Figure 1) [1]. As reported in several papers published in the 90’s, UDCA solubilizes cholesterol gallstones [2–3], it improves the liver function in cholestatic diseases [4–8] and it significantly decreases cholesterol saturation in the bile [8–10]. In terms of pharmacology, it is considered to be better than chenodeoxycholic acid (CDCA) in the treatment against biliary calculus, since it possesses high efficacy and total absence of side effects [11].

UDCA is commonly produced by transformation of cholic acid (CA), which is the most abundant and least expensive bile acid available. Because of the molecular complexity of bile acids, the chemical modification requires several protection and deprotection steps, resulting in an overall yield of about 30% [12–15]. For that reason, research has been performed on the development of more selective procedures which involve less reaction steps. In particular, microbial transformations [16–19] or chemoenzymatic procedures [20–21] employing CA, CDCA or lithocholic acid (LCA) as starting material have been studied.

This minireview summarises different aspects to be addressed and hurdles to be taken in the development of a selective and sustainable process for the production of UDCA. Different chemical, chemoenzymatic and enzymatic routes will be considered. In addition, the precursors availability as well as the substrate loading in the process and the requisites for potential new routes will be discussed. Furthermore, the potential benefits of a flow reactor setup for this multistep synthesis will be discussed.

## Review

### Precursor availability

#### Bile acids

The most important active ingredients of bile are the bile acids. Together with their salts, they allow the emulsification of lipids, a fundamental step for their absorption and digestion. Bile acids are 24-carbon containing 5β-steroids. Their structure contains multiple hydroxy substituents: the position and the stereochemistry of these OH groups influence the solubility and biochemical properties of the compounds. In CA, for example, the OH groups on the steroidal ring are all in α-position with respect to the ring plane, defining a structure in which the molecule has a polar and an apolar surface. For this reason, these molecules and their derivatives are defined as amphipathic. Bile acids are considered very important molecules for their ability to form micelles in an aqueous environment [22].

Bile acids biosynthesis takes place in the liver starting from cholesterol: 17 enzymes are involved in the production of these molecules. The final products are the so-called primary bile acids: CDCA and CA [23]. Subsequently, these bile acids can be modified by intestinal bacteria to form the secondary bile acids as, for example, deoxycholic acid (DCA), LCA and UDCA. Secondary bile acids can be subsequently resorbed and returned to the liver where they are re-secreted in a process known as enterohepatic circulation.

**Figure 2:**
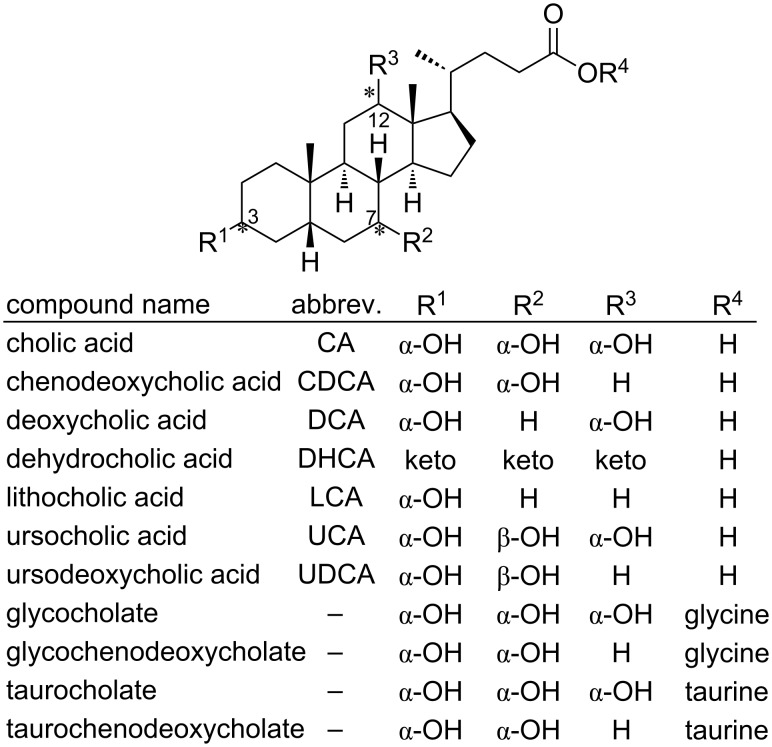
Chemical structures of bile acids and salts.

In mammals, bile acids are secreted as conjugated molecules with glycin or taurine (Figure 2), forming the so called bile salts, with slightly different properties (p*K*a, solubility) in comparison to the corresponding free acids [24–25]. These bile salts also lead to an increased retention in the intestine.

The only economically viable resource of bile acids is the bovine bile, which must be extracted at the time of slaughter.

In slaughterhouses, the bovine gallbladder is recovered during the processing of the meat and from a single cow, around 230 mL of bile can be obtained. The commercial prize of bile is in the range of 0.1–0.4 $/L. Bile acids represent roughly 0.7% (w/w) of the bile [26].

In order to extract and purify the different bile acids, bile is frozen and lyophilized: from 100 mL of bile 8 g of dry powder can be obtained. From these about 6.9 g of 90% pure bile acids can be obtained [27]. Cholesterol, cholesterol esters, triglycerides and free fatty acids are selectively extracted with organic solvents from aqueous buffers at different pHs. Then, the bile acids were separated from inorganic salts by extraction of the dry residue with absolute ethanol. The major components of the obtained mixture are the primary bile acids (CA and CDCA), secondary bile acids (DCA and LCA) and bile salts like taurocholic acid and glycocholic acid (derivatives of CA), taurochenodeoxycholic acid and glycochenodeoxycholic acid (derivatives of CDCA) and other conjugated salts of their 7-α-dehydroxylated derivatives [27].

#### CA

3α,7α,12α-Trihydroxy-5β-cholan-24-oic acid, also named cholic acid (CA, Figure 2), is one of the primary bile acids. It is almost insoluble in water, but soluble in methanol, ethanol and acetic acid. Differently from the acid, the sodium salt of CA is much more soluble in water at pH > 8.0 [28–29]. Salts of CA are called cholates. From the 3D structure of CA is it possible to observe an hydrophilic and an hydrophobic face, giving to CA its characteristic surfactant properties. CA is sold as a treatment for children and adults affected by bile acids synthesis disorders. Because of its abundance in bovine bile, CA is the precursor for UDCA.

#### CDCA

3α,7α-Dihydroxy-5β-cholan-24-oic acid, also known as CDCA (Figure 2), is a primary bile acid in human, but represents only 2.7% (w/w) of total bovine bile salts [30]: this is the main reason why it is not used as precursor for the preparation of UDCA.

CDCA can be used to treat gallstones avoiding, unlike CA, the downregulation of the cholesterol-7-α-hydroxylase, that represent the rate-limiting step in bile acid synthesis [31]. It can be metabolized by bacteria in the colon to form the secondary bile acid known as LCA [32].

#### LCA and other bile acids

LCA, also known as 3α-hydroxy-5β-cholan-24-oic acid (Figure 2), is a secondary bile acid. It is produced by bacteria in the colon from CDCA trough the dehydroxylation of the C7 functional group of the steroid framework. Low percentages of other secondary bile acids and related keto derivatives can be found in the bile. The solubility properties, interactions and metabolisms are related to the position and stereochemistry of the hydroxy groups attached to the steroid ring. A general structure with the names of several bile acids is reported in Figure 2.

#### Deconjugation

Free bile acids can be obtained from the corresponding bile amides (with glycine and taurine) through a deconjugation step. Chemically, the reaction is an hydrolysis of the amide derivatives, that can be carried out at high temperature in alkaline environment. This reaction requires large amounts of sodium hydroxide (30%) and high temperatures (120 °C) for extremely long times (8–12 hours). Few enzymes (acylases, EC: 3.5.1) have been reported to hydrolyse glycinates and taurinates to the corresponding carboxylic acid. Recently, Pedrini et al. [33] have isolated and characterized a cholylglycine hydrolase from *Xanthomonas maltophilia* CBS 827.97: this enzyme completely hydrolyses glycine and taurine conjugates in 20 minutes at 50 °C. Unfortunately the protein sequence of this enzyme is not reported, making recombinant expression and its industrial use impossible. A second enzyme, isolated in *Lactobacillus plantarum* and recombinantly expressed in *E. coli*, was reported by Christiaens et al. [34]: this enzyme shows almost the same properties of the one described above but with lower activities (the specific activities of *X. maltophilia* and *L. plantarum* acylases on glycocholic acid as substrate are 100 U/mg and 3.42 U/mg, respectively).

From a biocatalytic point of view, other acylases and the well-known lipases can be used to achieve the same reaction. Few literature reports can be found on the promiscuous amidase activity of wild-type or engineered lipases [35–36], but no one have tested their activities on bile salts.

#### Conclusions on the precursor availability

Natural UDCA is a very expensive pharmaceutical active ingredient since, to date, it can only be obtained by isolation from bear bile (practice used in traditional Chinese medicine) [37]. Alternatively, it can be produced by chemical transformation of CA and CDCA from the cheaper bovine bile. Nowadays, the transformation process is not yet optimized in terms of costs and environmental impact [20]. CA is the main constituent of bovine bile and is the main precursor for the synthesis of UDCA.

One of the main problems regarding the availability of precursors for the synthesis of UDCA is the direct relation with meat industries. The major manufacturers of bovine meat are in newly industrialised countries, in particular south America (Brasil) and India. Reports on Ecuadorian slaughterhouses [26,38–40], point out that in some cases there is a lack of adequate technical conditions and hygienic protocols, leading to environmental pollution and the need to include sanitary procedures in the processing of bile acids.

Alternative sources of sterols can be found in eukaryotic microorganisms like yeast and algae [41]. However, technological and scientific knowledge on these metabolic pathways are still in an early stage, and will not be included in this review.

### C12 Dehydroxylation

#### Chemical dehydroxylation

UDCA can be obtained by a multistep chemical synthesis starting from CA. Two main steps are involved: the dehydroxylation at C12 and the epimerization of the 7-OH group.

In order to achieve chemical dehydroxylation, firstly CA has to be oxidized in position C12 to the corresponding ketone, after which Wolff–Kishner reduction can be applied. This whole sequence comprises 5 steps [13]. After the protection of the carboxylic group by acid-catalyzed esterification (quantitative yield), the 3- and 7-OH groups are protected selectively with acetic anhydride and pyridine (yield 92%). The 12-OH group is oxidized with CrO_3_ (yield 98%) and, after a deprotection step in alkaline environment, the formed ketone group can be removed by a Wolff–Kishner reaction yielding CDCA (yield 82%). The overall yield of the dehydroxylation step is around 65%.

#### Wolff–Kishner reduction

**Figure 3:**
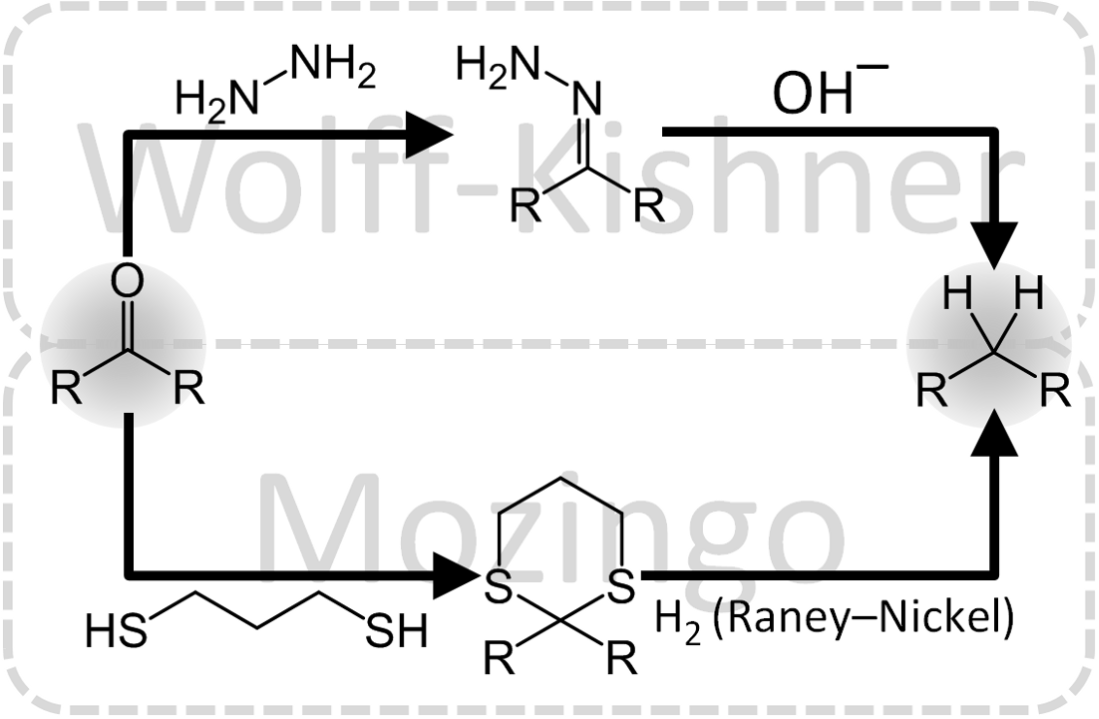
Comparison between Wolff–Kishner and Mozingo reduction. Notably the overall chemical reaction is the same for both cases.

The Wolff–Kishner reaction is widely used by chemists to remove carbonyl moieties yielding unsubstituted alkyl chains. The reaction requires two steps: the hydrazine first reacts with the ketone forming a hydrazone; The addition of a strong base and heat then promote a rearrangement with the elimination of N_2_ yielding the desired alkyl chain (Figure 3). This reaction is applied to the synthesis of UDCA in order to remove the carbonyl group at C12.

The use of hydrazine is a disadvantage in terms of safety (explosive risk) and economic costs (it should be used in excess relative to substrate). Several modifications of the Wolff–Kishner reaction can be found in literature: i.e., the Huang–Minlon modification, that consists of removing the unreacted hydrazine after the first step by distillation [42]. This results in higher yield and partial recovery of the unreacted hydrazine. In order to reduce the explosive risk, different hydrazine derivatives have been discovered and tested (e.g., methyl hydrazinocarboxylate, [43]). Other reports have demonstrated the feasibility of this reaction in a flow-system, avoiding the large excess of hydrazine, giving high yields in a more rapid reaction [44]. In this field, flow reactors offer benefits in terms of mass and heat transfer, both enhanced by the geometry of the reactor. Furthermore, the possibility to have a continuous production and to easily perform multiple modular reactions, leads to the improved scalability of these systems. In addition, a microwave-assisted Wolff–Kishner reduction has been examined with good results in a 30 seconds reaction [45]. Another option is the production of hydrazine in situ, using chemical methods. Also enzymatic activities towards hydrazine have been discovered [46], however, not enough optimized to be applicable.

#### A Wolff–Kishner alternative: the Mozingo reduction

The Mozingo reduction effects the same reaction as Wolff–Kishner, albeit under neutral conditions (Figure 3). It involves two steps: firstly, the carbonyl compound is converted into a dithioketal by adding a dithiol. The mechanism for this step is analogous to the mechanism for ketal or acetal formation except sulphur replaces oxygen as the nucleophile attacking the carbonyl group.

In a second step, the dithioketal is reduced to the corresponding methylene compound by hydrogenolysis in presence of Raney Nickel (actually used for the hydrogenation of fatty acids). In comparison to the Wolff–Kishner reaction, the use of hydrazine is replaced by the use of hydrogen gas, which can be seen as a double-bladed knife. The reduction step can also be performed with NaBH_4_ or other reductants. At the moment, a complete and clear reaction mechanism has not been well identified yet.

There is one report that suggests the application of this reaction for the synthesis of UDCA (yield 95%) [47]. The major problem related to this reaction is the very characteristic odor of ethanedithiol which is compared by many people to rotten cabbage. Ideally, there is the possibility of using other types of less volatile compounds but no reports thereof have been found.

#### 12α-Hydroxysteroid dehydrogenase

**Figure 4:**
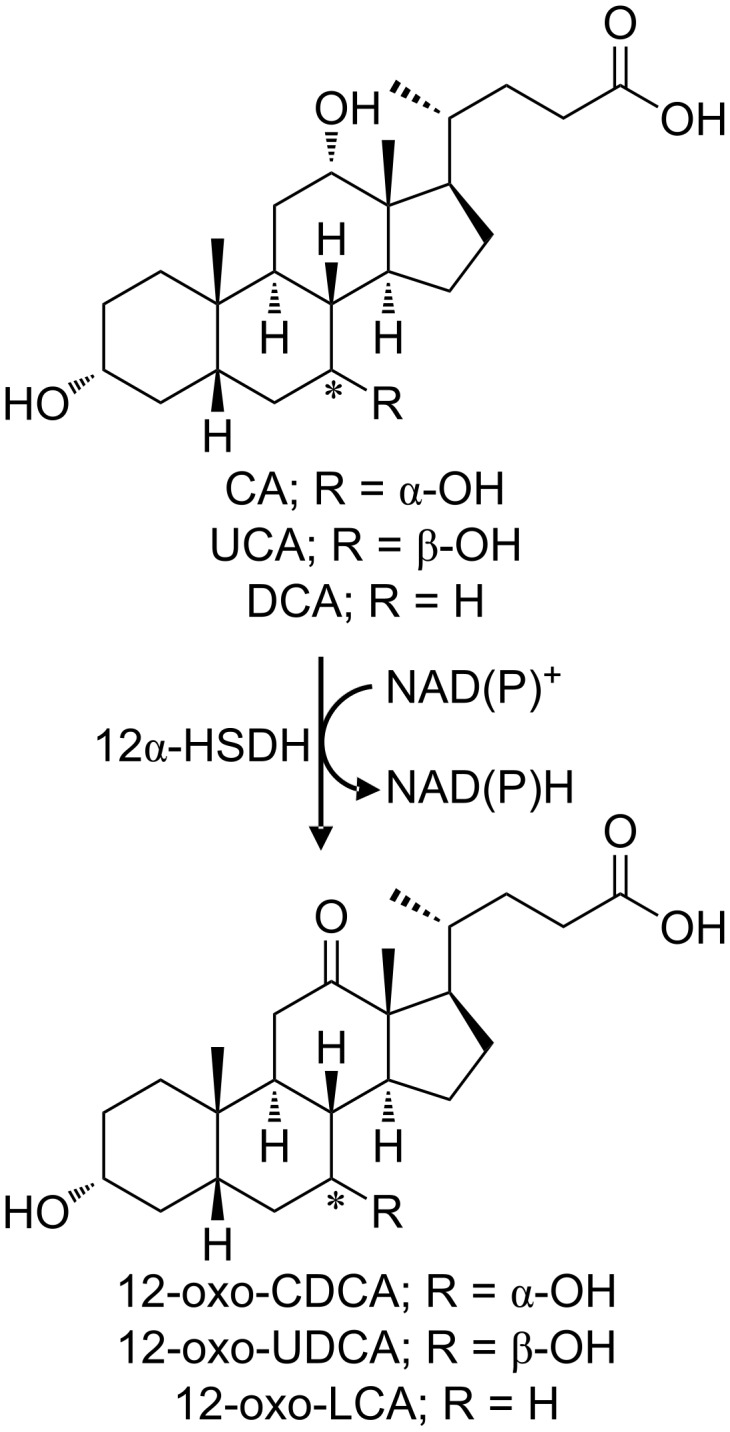
Reaction catalysed by the 12α-HSDH; the 12-OH group of CA or UCA is oxidized yielding 12-oxo-CDCA or 12-oxo-UDCA, respectively, with the concomitant reduction of one molecule of NAD(P)^+^.

12α-Hydroxysteroid dehydrogenases (12α-HSDH) are particularly interesting for the selective oxidation of the 12-hydroxy group of CA (Figure 4). These enzymes belong to the family of oxidoreductases with NAD^+^ or NADP^+^ as electron acceptor.

This oxidation is a mandatory step for removing the OH functionality at C12. In all the chemoenzymatic routes reported by Eggert et al. [20], the carbonyl group resulting from the oxidation of the 12-OH group was subsequently reduced by the Wolff–Kishner reaction (see above). The few cases showing a dehydroxylation in position C12 by bacteria are reported in the next paragraph. The NADP^+^-dependent 12α-HSDHs activity (Table 1, [48–51]) is spread among the strains of the genus *Clostridium* (e.g., *Clostridium leptum* [48] and *Clostridium* group P strain C 48–50 [49,52]). Furthermore, on the other side, the NAD^+^-dependent 12α-HSDHs activity was observed and reported in *Eubacterium lentum* and *Clostridium perfringens* [50–51]. A NAD^+^ dependent process for the production of 12-oxo-CDCA was patented in 2006 [53].

**Table 1:** Summary of reported 12α-HSDH. The given activity is based on CA as substrate.

microbial source	ref.	cofactor	specific activity	sequence

*Clostridium leptum*	[48]	NADP^+^	3.3 U/mg	/
*Clostridium* group P.	[49]	NADP^+^	128 U/mg	GenBank: HC036073.1
*Eubacterium lentum*	[50]	NAD^+^	0.5 U/mg	/
*Clostridium perfringens*	[51]	NAD^+^	/	/

#### Biocatalytic C12 dehydroxylation

In contrast to the reports on the epimerization of CA and CDCA with enzymes (see below ”Enzymes for the production of UDCA from CDCA”), the dehydroxylation of CA remains an undiscovered field for microbiologists and biochemists. To our knowledge, the only evidence of a bacterial-catalysed 12α-dehydroxylation was reported by Edenharder in 1983 [54]. He found eight strains of the *Bacteroides* genus that specifically dehydroxylate CA to CDCA. However, no other studies have been carried out concerning this topic. A method for the production of 12-dehydro steroids by the action of 12-dehydroxylase producing microorganism (*Clostridium perfringens* ATCC 19574) was patented in 1976 [55]. However, the presence and the expression of a protein that can catalyse this reaction were never confirmed in other papers.

The putative molecular mechanism for the C12 dehydroxylation is still unknown: it can resemble the dehydroxylation mechanism described for position C7 [56–58] (Supporting Information File 1, Figure S1). Interestingly all the genes catalysing that reaction where clustered in the BAI operon and, by analogy, it can be possible to design a biochemical pathway that specifically acts on C12 (see postulated sequence of steps in Supporting Information File 1, Figure S2). The reaction sequence can be divided in 3 steps: firstly, the substrate is oxidized by a specific alcohol dehydrogenase and an ene-reductase-like enzyme. Then dehydration occurs, catalysed by a specific dehydratase. The dehydrated product is then reduced through a 3-step-cascade reaction (catalysed by 3 different enzymes) giving the final dehydroxylated product.

### 7-OH epimerization: shift the equilibrium

#### Chemical epimerization of CDCA into UDCA

The second step of UDCA synthesis from CA, is the epimerization of the 7-OH group. Chemically, the 7α-OH group of CDCA, obtained by dehydroxylation of CA (see above “C12 dehydroxylation”), is selectively oxidized in the presence of sodium bromate [59] (yield 88%), *N*-bromosuccinimide [13,15] (ungiven yield) or 1-hydroxy-1,2-benziodoxol-3(1*H*)-one 1-oxide [60] (yield 90%) and subsequently reduced with metallic sodium in presence of imidazole and 1-propanol (yield 80%) yielding the 7β-OH epimer (UDCA) as imidazole salt. Notably, the regiospecific oxidoreduction of the 7α-OH group is achieved using weak oxidants: this behavior can be explained by the peculiar conformation of CDCA (the 7α-OH group is surrounded by alkyl chains, generating an hydrophobic environment that favors oxidation to the ketone, which is not the case for the other epimer). These data are supported by a density functional calculation or rather the differential change in electron density due to an infinitesimal change in the number of electrons [61]. The overall yield of the epimerization step is around 70% [12,15,62].

A further purification step is necessary for the preparation of free UDCA: it can be easily obtained with sequential esterification, extraction and hydrolysis (yield 91%). The theoretical yield of the whole process fluctuates around 30 to 40%.

#### Enzymes for the production of UDCA from CDCA

The enzymatic transformation of CDCA into UDCA can be obtained using different combinations of biocatalysts that act specifically on the 7-OH group of hydroxysteroids. In order to find the right combination of enzymes and the optimum reaction conditions, different aspects (enzymatic activities, equilibrium of the reaction, inhibition of enzymes by substrate and products and their stabilities) have to be assessed.

A list of enzymes that can be used for the transformation of CDCA is presented in the next paragraphs.

#### 7α-Hydroxysteroid dehydrogenases (7α-HSDH)

**Figure 5:**
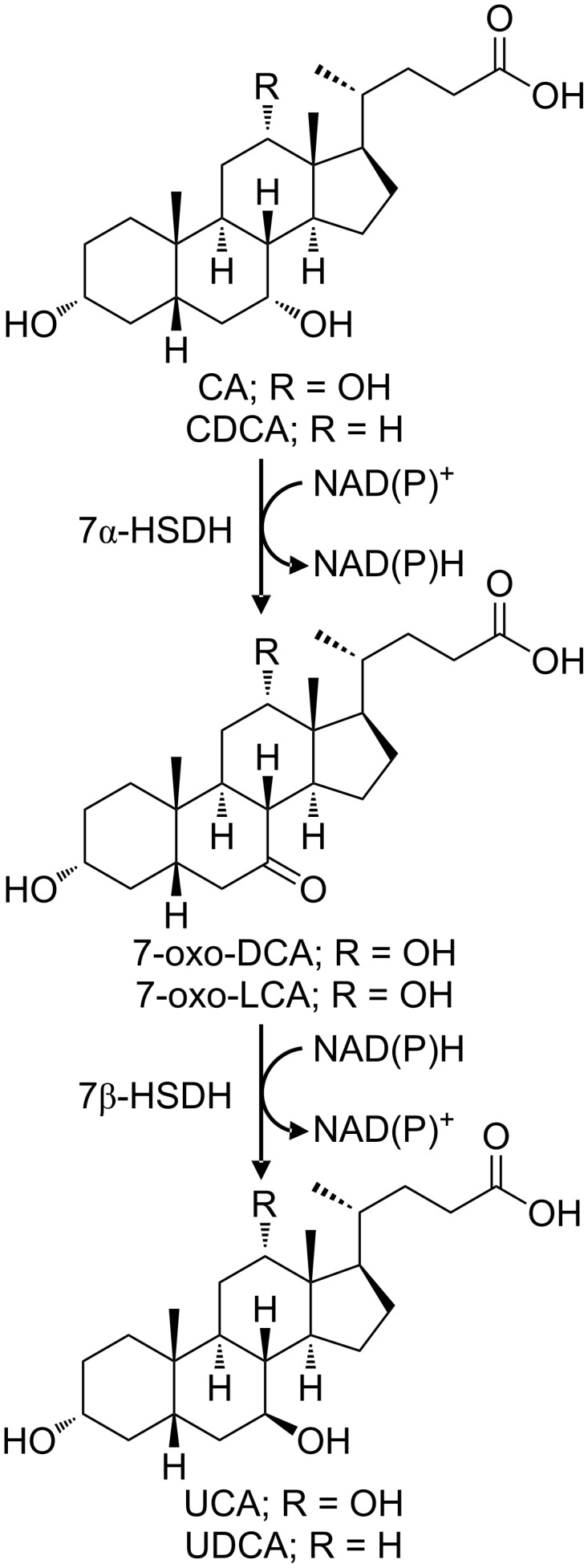
Epimerization reaction catalysed by the 7α-HSDH and 7β-HSDH; the 7α-OH group of CA (R = OH) or CDCA (R = H) is firstly oxidized by the 7α-HSDH yielding 7-oxo-DCA or 7-oxo-LCA, respectively. Subsequently, the keto group is reduced by the 7β-HSDH giving the final products UCA or UDCA.

These enzymes are able to oxidise specifically the α-hydroxy group at C7 together with the concomitant reduction of NAD^+^ or NADP^+^ (Figure 5). All of them are part of the group of the short chain dehydrogenases/reductases (SDR), showing a molecular weight around 30 kDa and a homodimeric or homotetrameric quaternary structure.

Reported 7α-HSDHs were isolated from both aerobic and anaerobic bacteria: the state of art, together with the cofactor dependence and the specific activities, are summarized in Table 2 [33,63–70]. Examples of processes for the selective oxidation of bile acids, their salts or derivatives were patented [71–72].

**Table 2:** Summary of reported 7α-HSDH. The given activity is on chenodeoxycholic acid as substrate.

microbial source	ref.	cofactor	specific activity	sequence

*Clostridium sordelii*	[63]	NADP^+^	1.1 U/mg	GenBank: AAA53556.1
*Eubacterium scindens*	[64]	NADP^+^	338 U/mg	GenBank: AAB61151.1
*Clostridium absonum*	[65]	NADP^+^	59 U/mg	GenBank: JN191345.1
*Clostridium difficile*	[66]	NADP^+^	8.5 U/mg	Genbank: YP 001086529
*Escherichia coli*	[67]	NAD^+^	190 U/mg	GenBank: KXH01569.1
*Pseudomonas* sp. B-0831	[68]	NAD^+^	941 U/mg	GenBank: D50325.1
*Bacteroides fragilis*	[69]	NAD^+^	351 U/mg	GenBank: OGX95366.1
*Xanthomonas maltophilia*	[33]	NAD^+^	70 U/mg	/
*Comamonas testosteroni*	[70]	/	/	/

In addition to these reported biotransformations, many additional 7α-HSDHs have been discovered and reported over the past years. About 500 entries can be found in Reference sequence (RefSeq) database at NCBI [73] typing “7-alpha-hydroxysteroid dehydrogenase”.

#### 7β-Hydroxysteroid dehydrogenases (7β-HSDH)

Unlike their homologues, only few examples of bioconversion with 7β-HSDH have been reported in literature (Table 3, [33,65,74–77], Figure 5): one NADP^+^-dependent dehydrogenase from *Clostridium absonum* [65] was used in two different processes for the production of UDCA [78–79]. The NADP^+^-dependent enzyme from *Eubacterium aerofaciens* shows a significantly lower specific activity [74] and another NADP^+^-dependent enzyme was isolated from *Ruminococcus gnavus* [75]. In order to increase the activity and stability of 7β-HSDHs, protein engineering studies were carried out, as described in literature by Weuster-Botz et al. [77] and Zheng et al. [76]. Up till now, *Xanthomonas maltophilia* 7β-HSDH (33 U/mg) represents the only isolated NAD^+^-dependent enzyme [33]. Unfortunately its protein sequence has not been reported.

**Table 3:** Summary of reported 7β-HSDH. The given activity is based on 7-oxo-LCA as substrate.

microbial source	ref.	cofactor	specific activity	sequence

*Clostridium absonum*	[65]	NADP^+^	65 U/mg	GenBank: JN191345.1
*Eubacterium aereofaciens*	[74]	NADP^+^	30 U/mg	GenBank: ZP0177306.1
*Ruminococcus gnavus*	[75]	NADP^+^	23 U/mg	GenBank: ZP02041813
*Collinsella aerofaciens*	[77]	NADP^+^	15 U/mg	GenBank: WP006236005
*Collinsella aerofaciens*	[77]	NADP^+^	21 U/mg	Engineered^a^
*Ruminococcus torques*	[76]	NADP^+^	8.6 U/mg	GenBank: WP015528793
*Ruminococcus torques*	[76]	NADP^+^	46.8 U/mg	Engineered^b^
*Xanthomonas maltophilia*	[33]	NAD^+^	33 U/mg	/

^a^G39A variant of the 7β-HSDH from *Collinsella aerofaciens*; ^b^T198V/V207M variant of the 7β-HSDH from *Ruminococcus torques.*

#### Biocatalytic processes

Both microorganisms and purified enzymes have been applied for the fully biocatalytic epimerization of the 7-OH group. Several examples reported in literature are summarized in Table 4 [80–85].

**Table 4:** Summary of reported whole-cell transformations with wild type microorganisms. Epimerization yields of CDCA to UDCA are given.

microorganism	ref.	yield (%)

*Colinsiella aerofaciens*	[80]	–
*Clostridium absonum*	[81]	75%
*E. coli + Bacteroides fragilis*	[82]	25–30%
*Colinsiella aerofaciens + Bacteroides fragilis*	[82]	95%
mixed culture	[83]	–
*Clostridium limosum*	[84]	55–60% (75–80%^a^)
*Stenotrophomonas maltophilia*	[85]	27% (80%^b^)

^a^Reported yield of epimerization of CA to ursocholic acid; ^b^reported yield of epimerization of 12-oxo-CDCA to 12-oxo-UDCA.

The use of whole-cell conversion offers both advantages and disadvantages: wild-type microorganisms are normally difficult to grow, especially if the enzyme expression is related to anaerobic conditions. In addition the pathogenicity of these microorganisms represents a problem for their use in the pharmaceutical industry; additional steps of purification and control of sterility are necessary to obtain a safe product for the market. Otherwise, the circumvention of protein isolation and production makes it cheaper than their free-enzyme analogues.

In this way, the use of lyophilized whole-cell containing recombinant HSDHs can represent a solution in the reduction of catalyst costs, maintaining a reasonable safety. This approach was followed by Braun et al. and Sun et al. obtaining the 12-oxo-UDCA with a yield of 99.5% using engineered *E. coli* cells [86–87]. In comparison to the systems that employed purified enzymes (see below), this approach allows higher substrate loading (70–100 mM).

**Figure 6:**
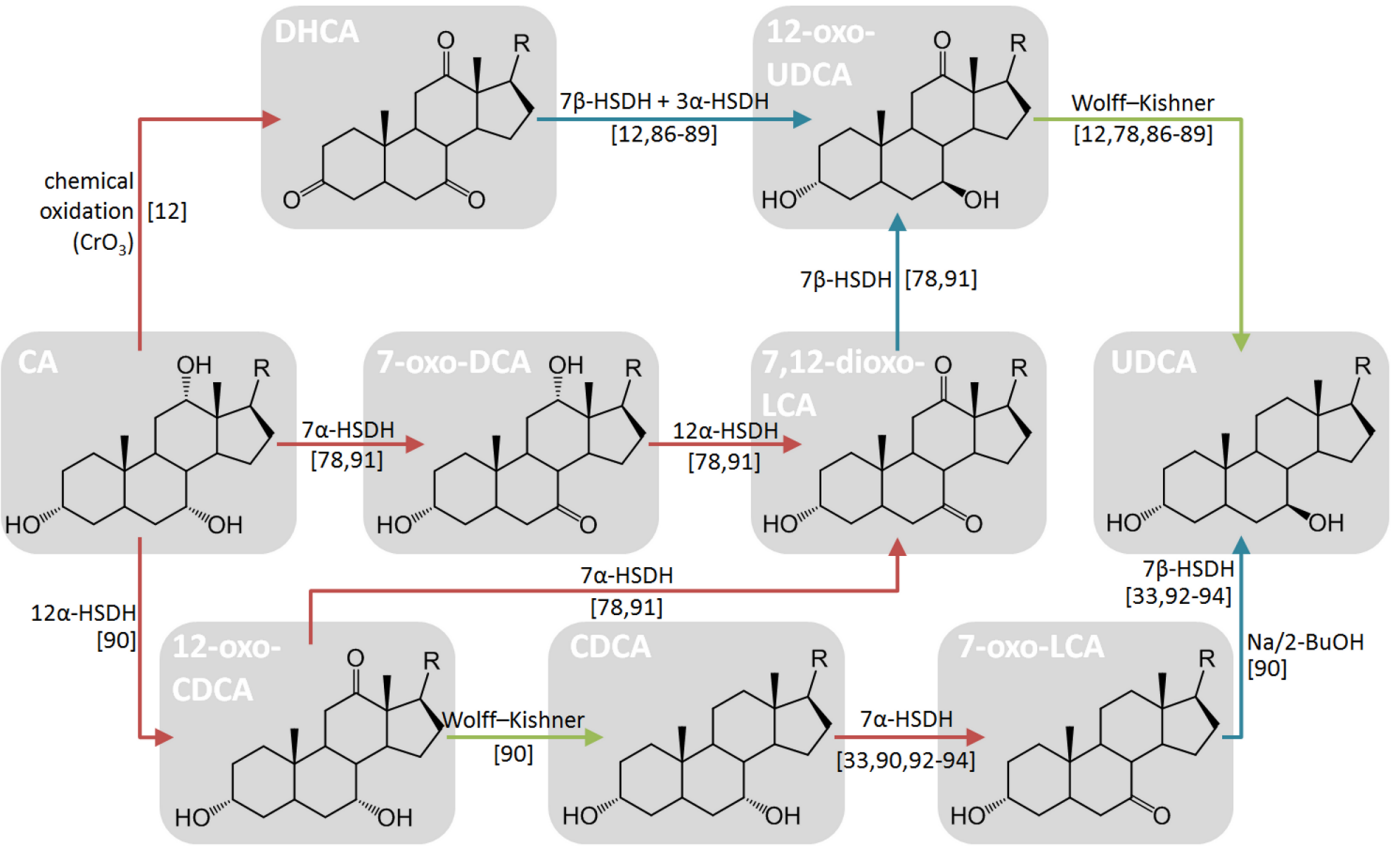
Overview of the chemoenzymatic process for the production of UDCA from CA: The oxidation, reduction and dehydroxylation reactions are highlighted with a red, blue and green arrow, respectively. Names of compounds are indicated in the box. The general stereoinformation of steroid scaffold is shown in Figure 2. R = 4-pentanoic acid.

Several enzymatic systems have been proposed in the literature, together with cofactor regeneration systems. As general rule, the oxidative and reductive steps are coupled with a related regeneration system. In this way, the equilibrium of the reaction can be pushed to the production of UDCA. An overview of reported enzymatic and chemoenzymatic cascades is summarized in Table 5 and Figure 6 [12,33,78,86–94].

**Table 5:** Summary of reported chemoenzymatic transformations with purified enzymes.

reaction pathway	ref.	conversion yield (%)	productivity (g L^−1^ d^−1^)

DHCA→12-oxo-UDCA	[12]	85%	7.0
	[86]	95%	9.6
	[87]	99%	40.1
	[88]	95%	55.6
	[89]	99%	7.3

CA→UDCA	[90]	70%	1.8

CA→12-oxo-UDCA	[78]	88%	8.0
	[91]	73%	24.1

CDCA→UDCA	[33]	82%	0.02
	[92]	100%	47.2
	[93]	100%	88.5
	[94]	63%	3.0

From the comparison of the different routes, it can be observed that higher productions are obtained when the Wolff–Kishner reaction is carried out in the late stage. The typical substrate loading in systems employing purified enzymes is in the range of 10–15 mM. This disadvantage is partially compensated by the reuse of the biocatalysts through the employment of membrane reactors [12,78] or the immobilization of enzymes [93]. The first system shows high stability (enzymes in the membrane reactor have a half-life of 1–2 weeks) and the biocatalysts can be reused for eight cycles of conversions. On the other hand, immobilized enzymes show a higher productivity (88.5 vs 8 g L^−1^ d^−1^) despite the fact that the half-life (23 h) is lower and the biocatalyst can be reused for only five cycles of conversions.

**Figure 7:**
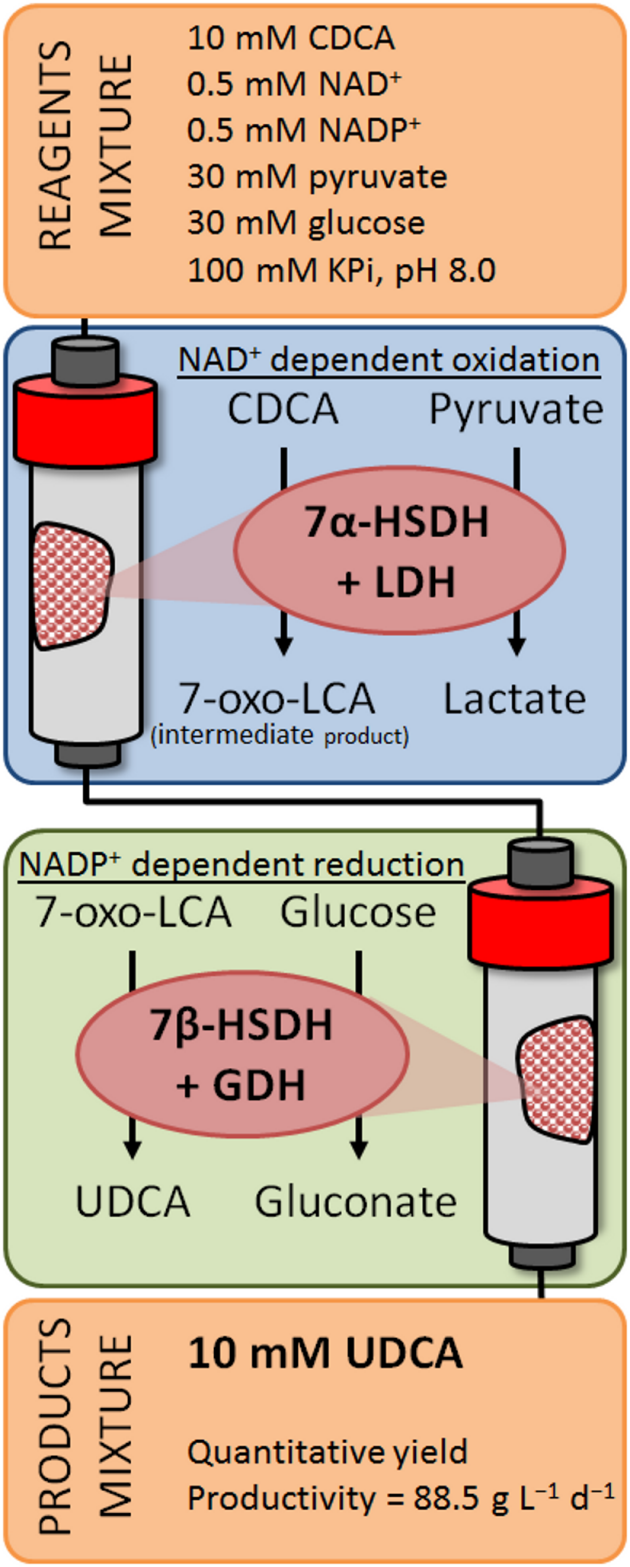
Schematic representation of the flow reactor for the continuous conversion of CDCA to UDCA [93].

In order to reduce the mechanical stress that might inactivate the immobilized enzymes, the flow-system represents a valid technology. The packed-bed reactor set up by Zheng et al. partially solved this problem, achieving full conversion of CDCA into UDCA for at least 12 hours. This represents an improvement compared to the use of the same biocatalysts under batch conditions. This particular flow-system consists of two modular column reactors (Figure 7): firstly, CDCA is oxidized to 7-oxo-LCA by an immobilized NAD^+^-dependent 7α-HSHS (first reactor column); afterwards, 7-oxo-LCA is reduced to UDCA by an immobilized NADP^+^-dependent 7β-HSDH (second reactor column). The cofactors are individually regenerated in each column by the co-immobilized enzymes, lactate dehydrogenase (LDH) and glucose dehydrogenase (GDH), respectively.

The decoupling of the 2 reactions is an elegant way to spin the equilibrium but, in every catalytic cycle, the co-substrates used to regenerate the cofactor have to be added in great surplus, leading to additional costs and additional problems in the downstream process. The most used enzymes for the cofactor regeneration are glucose dehydrogenase (glucose to glucuronic acid), lactate dehydrogenase (pyruvate to lactate), glutamate dehydrogenase (α-ketoglutarate to glutamate) and formate dehydrogenase (formate to CO_2_). In particular, the last enzyme is interesting because formate is cheap and, because of the gaseous nature of CO_2_ as product, the equilibrium of the reaction is entropically favoured.

Pedrini et al. in 2006 [33] reported the successful epimerization of CDCA to UDCA using a redox-neutral cascade reaction, with two NAD^+^-dependent dehydrogenases. In this way the requirement of external systems for cofactor regeneration was circumvented and UDCA was obtained with a final yield of 75%. Interestingly, the addition of 2-hexanol led to an increase of NADH available for the reduction of 7-oxo-LCA and a final yield of 82% was observed. According to the authors, the presence of another alcohol dehydrogenase in the partially-purified enzyme preparation increases the amount of NADH for the 7-oxo-LCA reduction.

To conclude, it is difficult to denote the “best” route for the 7-OH epimerization. All the processes mentioned, demonstrate reasonable yield and high selectivity. A redox-neutral cascade seems most elegant, but in order to fully understand and push the equilibrium of the reaction, a full biochemical characterization and a deep knowledge of the kinetics and stability of the involved enzymes is required.

#### Other ways to obtain 7-OH epimerization

Other chemical routes for the production of UDCA have been patented and published: for example, Dangate et al. [60] proposed a chemical route where the order of the two steps (C12 dehydroxylation and C7 epimerization) is reverted and the specific oxidation of 7- and 12-OH group can be achieved without any protection step (yield 53%).

Another interesting chemoenzymatic way to obtain the epimerization of the 7-OH group consists is the removal of the functionality and the subsequent rehydroxylation with a specific final chiral configuration. Both steps can be performed by enzymes and/or microorganisms: Sawada et al. [95] reported that a fungal strain (*Fusarium equiseti* M41) was able to introduce a 7β-hydroxy group into LCA by hydroxylation forming UDCA directly.

Later, many other microorganisms with a 7β-hydroxylating activity were discovered in strains of actinobacteria and ﬁlamentous fungi [96–97]. The key-enzyme in that pathway is a P450-like enzyme that catalyses the specific and irreversible 7β-hydroxylation. On this topic, a recent work by Kollerov et al. [98] describes several DCA modifying filamentous fungi strains (mostly ascomycetes and zygomycetes): the highest 7β-hydroxylase activity level was found in *Fusarium merismoides* VKM F-2310.

**Figure 8:**
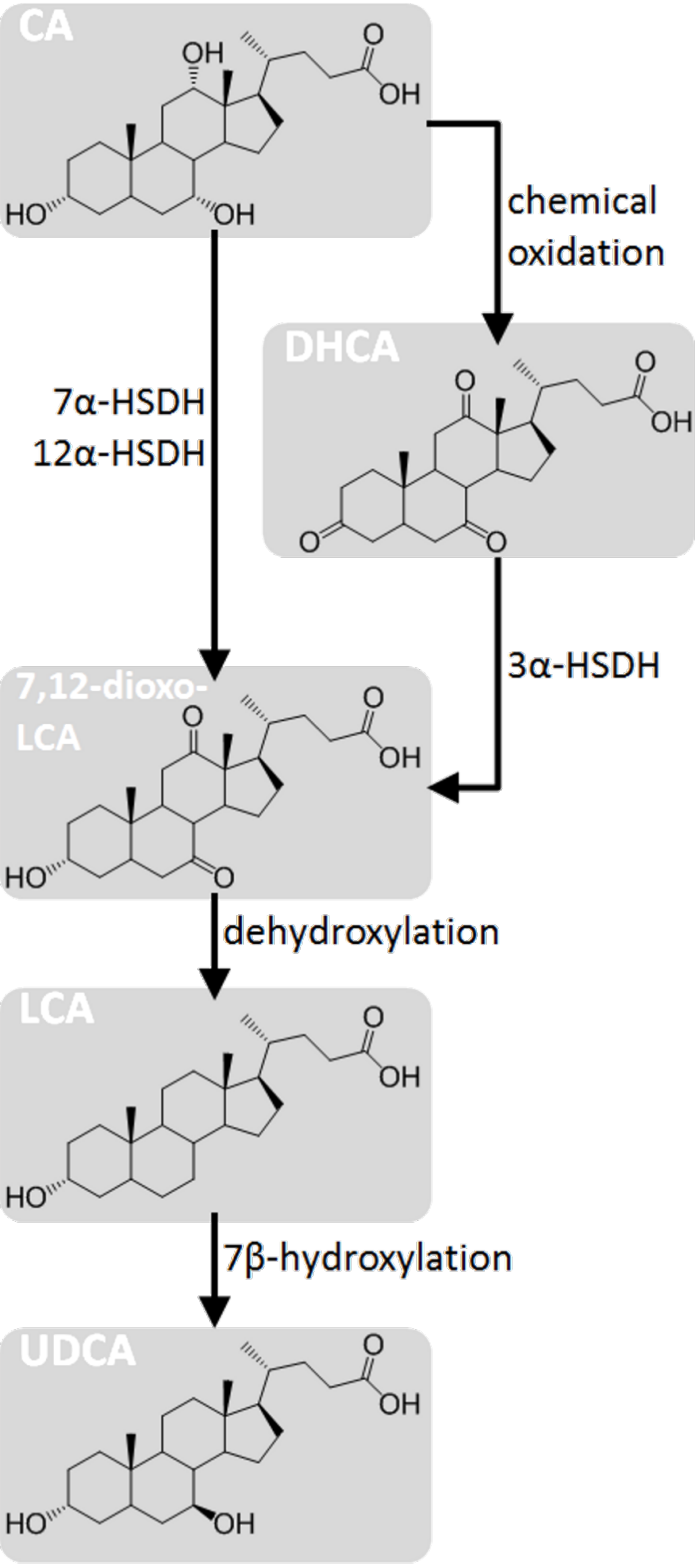
Chemoenzymatic pathways for the formation of UDCA from CA that profit by the C7 hydroxylation activity described by Sawada et al. [95]. CA can be transformed to 7,12-dioxo-LCA trough specific oxidation of 7α-OH and 12α-OH with a 7α-HSDH and 12α-HSDH, respectively. Alternatively, 7,12-dioxo-LCA can be obtained by chemically oxidizing (e.g., with CrO_3_) all the hydroxy groups, yielding DHCA and then reducing the 3-oxo group to 3α-OH by a 3α-HSDH. LCA can be obtained from 7,12-dioxo-LCA through dehydroxylation by Wolff–Kishner or Mozingo reduction. Finally, UDCA can be obtained from LCA by 7β-hydroxylation. The general stereoinformation of the steroid scaffold is shown in Figure 2.

The possibility to access that kind of chemical and chemo-enzymatic reactions pave the way for the design of other unexplored routes for the production of UDCA (example in Figure 8).

In addition, other reported enzymes can eventually play a role in the cascade reaction synthesis of UDCA. For example the 3α-HSDHs [51,99] catalyze the oxidoreduction of the 3α-OH groups to the corresponding ketones and the well-known laccase-TEMPO system [100] can be used for the unselective oxidation of CA to dehydrocholic acid (DHCA).

### Solvent and substrate loading considerations in processing

For an economically and environmentally sustainable process volumetric productivities have to be considered. In other words substrate loadings cannot be too low. While it does not represent a problem in chemical synthesis (UDCA, CDCA and CA are pretty soluble in alcohols like methanol and ethanol), the water-based environment required by enzymes is an obstacle in the development of a biocatalytic process.

In comparison to CA, the solubility of CDCA and UDCA at pH 8.0 (typically used for HSDHs) is lower (around 25 mM) [101], and it could be increased when adding methanol or ethanol as co-solvent. In addition, at higher concentrations than the critical micelles concentration (CMC), bile acids tend to form micelles: this phenomenon, due to the amphipathic structure of these molecules, is limiting the availability of free hydroxysteroids in solution. The reported CMCs of bile acids are in the range of 5–15 mM [101–104]. Accordingly, the addition of co-solvents increases the CMC of bile acids and the availability of monomers in solution. Notably, HSDHs are relatively stable and active in 10–20% methanol. Moreover, the immobilization of the enzyme can provide a higher stability to the protein and makes the system work also at higher concentrations of co-solvent [105]. However, working with a diluted solution, produce a large amount of wastewater that had to be treated.

Another option is represented by biphasic systems: In these cases, the organic phase works as reservoir of reagents and products. This methodology is widely used in biocatalysis to solve solubility issues. Unfortunately, the solubility of hydroxysteroids in non-alcoholic organic solvents (e.g., ethers, alkanes, dichloromethane, chloroform) is not very high (e.g., the reported solubility values for CDCA and CA in chloroform are 7.6 and 14.4 mM, respectively [102]). Several attempts to carry out hydroxysteroid transformations in biphasic systems were reported [106–108]: good conversions and an increase of reaction rates were observed for 7-OH and 3-OH epimerizations. In these cases the reactions were carried out at final concentrations of substrate in the range of 10–20 mM, the usual substrate loading in monophasic systems.

Of no lesser importance, the increased amount of substrates and products up to relevant concentrations for industrial application can inhibit the enzymes used in the biocatalytic process. Several examples are reported in literature about substrate or product inhibition of HSDHs. Protein engineering could help to solve or lowering the effect of these issues, leading to the optimization of the biocatalyst for industrial applications. In addition, the use of flow-reactors can be beneficial to diminish substrate and product inhibition by controlling the contact time.

In conclusion, the increase of the substrate loading is one of the main challenges in the development of an efficient biocatalytic system for the production of UDCA form CA. More research is needed to address this aspects.

## Conclusion

The organic synthesis of CDCA and UDCA starting from taurinated and glycinated cholic acid is a long process, complicated and risky due to the nature and toxicity of the reagents used, the costs of disposal of large amounts of sodium hydroxide, chromium salts and organic solvents, and the purification processes necessary to eliminate byproducts formed at each step of reaction involved. All this extends the time, increases costs and decreases production yields. Therefore, research nowadays is geared towards more economical synthesis methods that are waste-free and safe to operate.

An approach that bears great promise is the biotransformation with non-pathogenic, easy-to-manage microorganisms, and their enzymes. Several chemical, chemoenzymatic and enzymatic routes have been proposed for the production of UDCA. In view of sustainability, instead of pursuing a step-wise approach, an integrated one-pot or one-flow reaction, involving highly selective enzymatic steps would be preferred.

When a multi-enzyme system is employed, the different enzyme activities, pH optima, cross reactions and inhibitions have to be taken into account in order to reach high product yields [109–111]. Furthermore, when a combination of chemical and enzymatic steps is employed special attention has to be paid to the compatibility: the combination of enzymatic and chemical transformation steps is the main task to achieve in order to obtain high yields of UDCA.

Nowadays, the most promising system for the biocatalytic production of UDCA are flow-reactors. They can be used for the setup of continuous working systems, lowering the quantity of catalyst needed and the time of each reaction. This technology was recently employed by Zheng et al. [93] leading to high yields (99%) and productivity (88.5 g L^−1^ d^−1^) for the epimerization of CDCA to UDCA. However, the employed enzymes have different cofactor specificities, leading to the consumption of stoichiometric amounts of sacrificial substrates (pyruvate and glucose). In addition, substrate loadings in the latter process are still modest (10 mM). Therefore, there is much room for improvement and further studies are needed to design a truly sustainable integrated process for the production of UDCA.

## Abbreviations

CA, cholic acid; CDCA, chenodeoxycholic acid; DCA, deoxycholic acid; DHCA, dehydrocholic acid; LCA, lithocholic acid; UCA, ursocholic acid; UDCA, ursodeoxycholic acid; HSDH, hydroxysteroid dehydrogenase; LDH, lactate dehydrogenase; GDH, glucose dehydrogenase.

## Supporting Information

Supporting Information features Figure S1 relative to the C7 dehydroxylation mechanism of hydroxysteroids and Figure S2 relative to the postulated biochemical pathway for the C12 dehydroxylation.

File 1 Supporting Figures S1 and S2.
