# Supplementary material for: Latest development in the synthesis of ursodeoxycholic acid (UDCA): a critical review
Source: Beilstein J Org Chem. 2018 Feb 20;14:470–83. doi: 10.3762/bjoc.14.33 (PMC5827811; doi:10.3762/bjoc.14.33)
Supplement: File 1 — Supporting Figures S1 and S2. [file Beilstein_J_Org_Chem-14-470-s001.pdf]

Supporting Information

for

Latest development in the synthesis of ursodeoxycholic acid (UDCA): a critical review

Fabio Tonin and Isabel W. C. E. Arends*

Address: Department of Biotechnology, Delft University of Technology, Van der Maasweg 9, 2629 HZ Delft, The Netherlands

Email: Isabel W.C.E. Arends - I.W.C.E.Arends@tudelft.nl

*Corresponding author

Supporting Figures S1 and S2

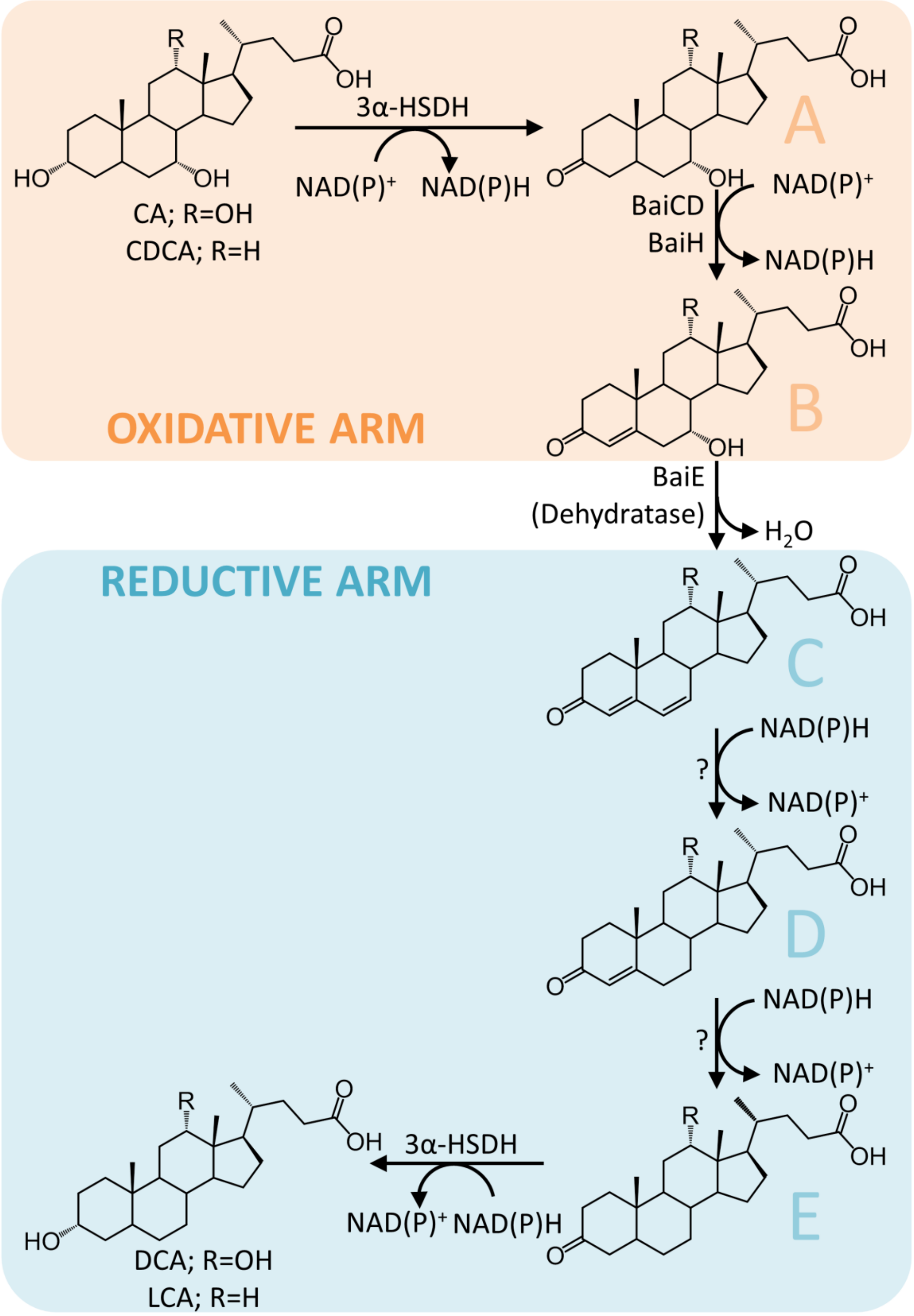

Figure S1: Proposed pathway involved in the synthesis of secondary bile acids. The oxidative and reductive arms are highlighted in orange and blue boxes, respectively. CA is oxidized first to (A) 3-oxo-CA and subsequently to (B) 3-oxo-Δ^4-CA. The latter compound is dehydrated by BaiE yielding (C) 3-oxo-$\Delta^{4,6}$-DCA and furtherly reduced to (D) 3-oxo-Δ^4-DCA, (E) 3-oxo-DCA and DCA. If CDCA is employed the names of the intermediates are: (A) 3-oxo-CDCA, (B) 3-oxo-Δ^4-CDCA, (C) 3-oxo-$\Delta^{4,6}$-LCA, (D) 3-oxo-Δ^4-LCA and (E) 3-oxo-LCA. The name of known enzymes are given; hypothetical enzymes not yet identified are marked with a question mark. The reaction from the compound D to E can be catalyzed by the same enzymes used for the oxidation (BaiCD or BaiH).

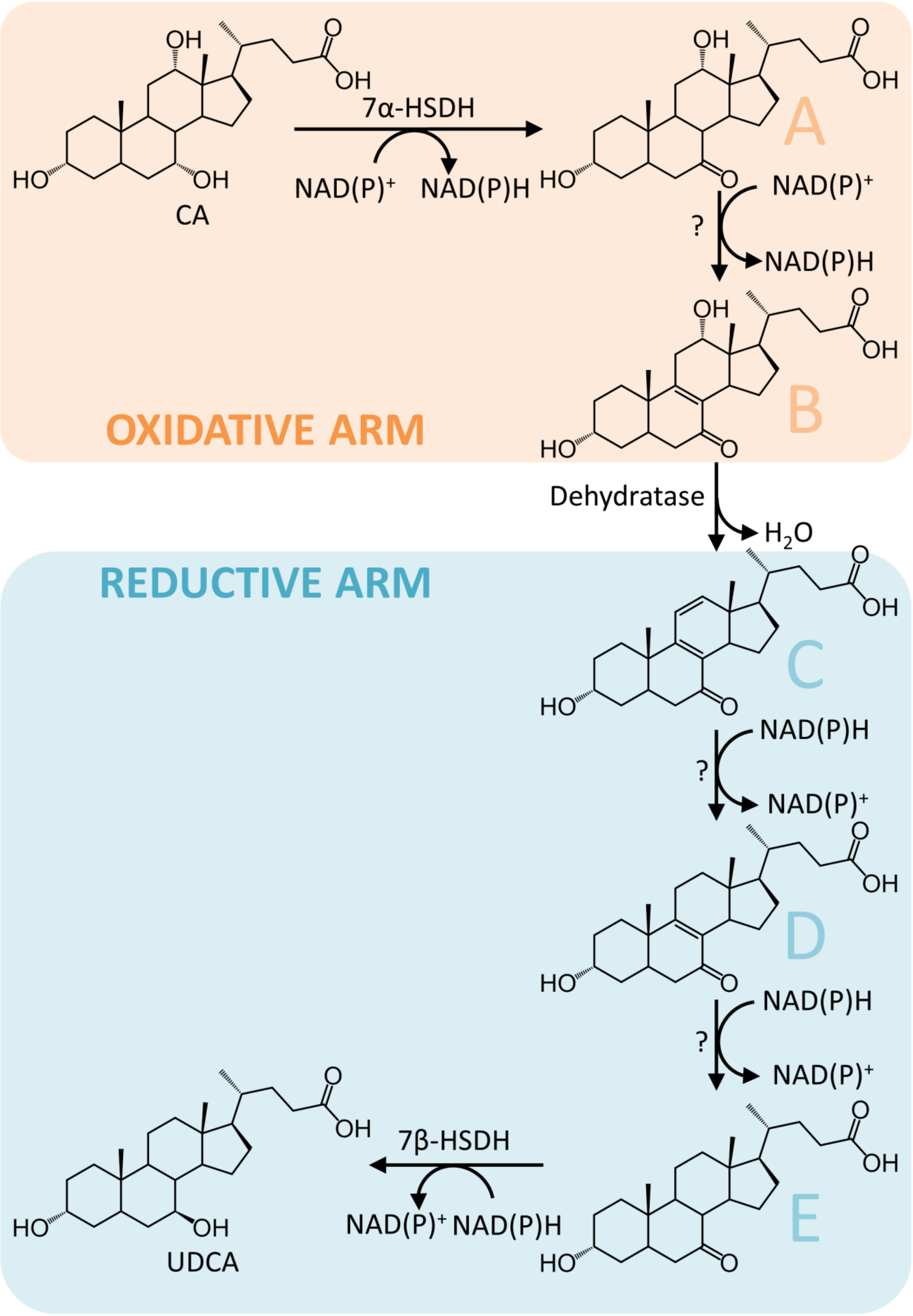

Figure S2: Proposed mechanism for the C-12 dehydroxylation designed by analogy to the C7 one. Also in this case, the oxidative and reductive arms are highlighted in orange and blue boxes, respectively. (A) 7-oxo-DCA, (B) 7-oxo-Δ^8-DCA, (C) 7-oxo-$\Delta^{4,11}$-LCA, (D) 7-oxo-Δ^8-LCA and (E) 7-oxo-LCA
